# 

**Published:** 1896-01

**Authors:** 


					﻿Vol. XVI.
JANUARY, 1896.
NO. 1.
THE
pOMEOPATHlC PHYSICIAN,
A Monthly Journal of Homoeopathic Materia Medica
and Clinical Medicine.
“ He it a freeman whom truth makes free, and all are slaves betide."
"Seek the Truth: come whence it may, cost what it will."
EDITED AND PUBLISHED BY
WALTER M. JAMES, M. D.
PHILADELPHIA:
JSTo. 1231 LOCUST STREET.
LONDON:
ALFRED HEATH & CO., 8ole Agents fob Gbeat Bbitain,"-
114 Ebury Street, Eaton Square.1.1	£
Fecrrit/ Subscription, fSJtO, insariablg in advance. Single numbers, 9S els.
Entered at the Philadelphia Port-OSeejM Second-Cl a*» Mall Matter.
				

